# P-179. A Retrospective Review of *Plasmodium falciparum* Malaria at a Pediatric Hospital in the United States

**DOI:** 10.1093/ofid/ofae631.384

**Published:** 2025-01-29

**Authors:** Shreya M Doshi, Emily Ansusinha, Aimee Dassner, Alexandra B Yonts, Barbara A Jantausch

**Affiliations:** Children's National Health System, Washington DC, District of Columbia; Children's National Hospital, Washington, District of Columbia; Children's National Health System, Washington DC, District of Columbia; Children's National Hospital/ George Washington University, Washington, District of Columbia; Children's National Hospital, Washington, District of Columbia

## Abstract

**Background:**

Malaria infects millions globally, and recently is also reported to have endemic transmission in the US. CDC definition of severe malaria applies to patients with at least one of the following : High percent parasitemia (≥5%), impaired consciousness, seizures or acute respiratory distress syndrome (ARDS), acidosis, acute kidney injury (AKI), DIC, jaundice, (Hb < 7 g/dL ). Artesunate was recently approved in 2020 for treatment of severe malaria. Our goal was to describe demographic features, insurance status, clinical characteristics, and hospital course of children with severe malaria admitted to our hospital from 01/2018-11/2023 and assess any differences in outcomes between those receiving quinidine versus artesunate.

**Figure:**
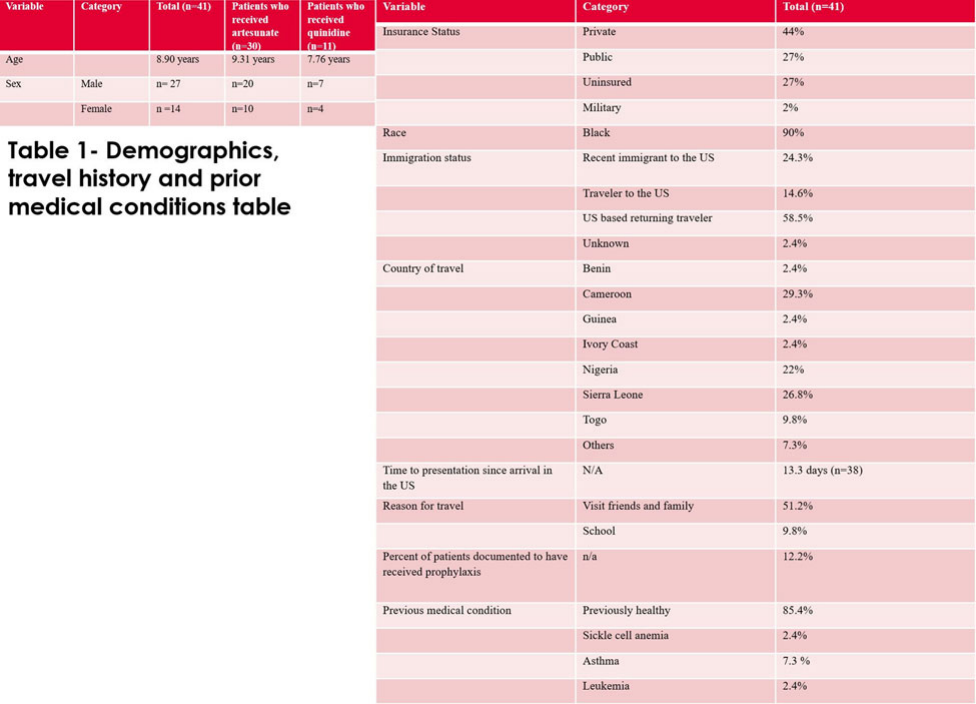

**Methods:**

After IRB approval medical records of children with severe malaria due to *P. falciparum* were reviewed for demographic and clinical characteristics of patients. Time to reduction in parasitemia (less than 1%) after drug administration patients who received artesunate (n=30) & patients who received quinidine (n=11) was calculated. Readmission to the hospital for relapsed of malaria or side effects, including post-artesunate delayed hemolysis was noted.

**Figure:**
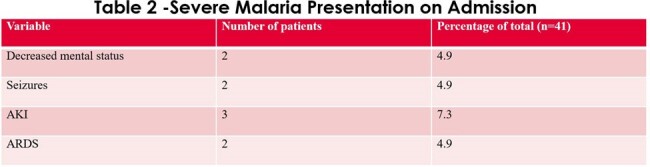

**Results:**

In an analysis of 41 patients with severe malaria 27 % were uninsured, 24 % were recent immigrants to the US. Peak parasitemia was mean was 11.2 %. Time to reduction in parasitemia on artesunate (n=30) was 24.2 hours and time to reduction in parasitemia on quinidine (n=5) was 23.8 hours (comparable). See Tables 1-3, and Figure 1 for results.

Table 3

**Figure:**
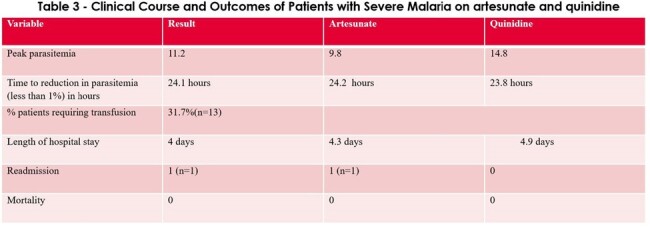

**Conclusion:**

Severe malaria presentation in children is unique to travelers and immigrants. Clinical presentation and features are important to know as it could have fatality. Time to reduction in parasitemia was comparable for the two drugs. Artesunate was well tolerated with low incidence of post-artesunate delayed hemolysis (n=1) leading to readmission. No deaths were attributable to malaria. Research and advocacy are important as 27 % were uninsured, 24 % were recent immigrants to the US highlighting the need for systemic changes. Future studies could include time to drug administration and time to reduction in parasitemia for children in the US vs children in developing countries highlighting access and equity issues globally.

Figure 1

**Figure:**
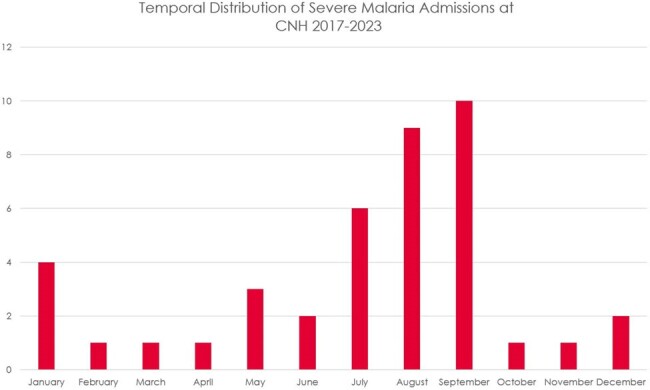

**Disclosures:**

**Alexandra B. Yonts, MD**, Pfizer: Grant/Research Support

